# Effect of Different Disinfection Procedures on the Microbiological Quality and Germination Efficacy of Sprouted Quinoa (*Chenopodium quinoa*) Flour

**DOI:** 10.3390/foods14183196

**Published:** 2025-09-13

**Authors:** Silvia Melissa García-Torres, José António Teixeira, Christian R. Encina-Zelada, Cristina L. M. Silva, Ana Maria Gomes

**Affiliations:** 1 CBQF—Centro de Biotecnologia e Química Fina—Laboratório Associado, Escola Superior de Biotecnologia, Universidade Católica Portuguesa, Rua Diogo Botelho 1327, 4169-005 Porto, Portugal; s-smgtorres@ucp.pt (S.M.G.-T.); clsilva@ucp.pt (C.L.M.S.); 2 Departamento de Tecnología de Alimentos, Facultad de Industrias Alimentarias, Universidad Nacional Agraria La Molina, Av. La Molina s/n Lima 12, Lima 15024, Peru; cencina@lamolina.edu.pe; 3 Center of Biological Engineering, School of Engineering, University of Minho, Gualtar Campus, 4704-553 Braga, Portugal; jateixeira@deb.uminho.pt

**Keywords:** mesophilic aerobic bacteria, enterobacteria, ultrasound, combined methods

## Abstract

Grain processing requires sustainable and innovative alternatives. Germination, which enhances the nutritional value of grains, can also increase the microbial load, posing a challenge to food safety. In quinoa, a superfood with an exceptional nutritional profile, germination could maximize its benefits if proper methods are applied to ensure safety. The effects of different disinfection methods on mesophilic aerobic bacteria, enterobacteria, and the germination capacity of two Peruvian quinoa varieties (Rosada de Huancayo (RH) and Pasankalla (PK)) were evaluated for germinated flour production. Seven treatments were applied: four with chemical agents (200 ppm sodium hypochlorite, 4% acetic acid, 8% H_2_O_2_, and 70% alcohol) and three combined methods (sodium hypochlorite with ultrasound (35 kHz, 15 or 30 min), and hot water (50 °C) with H_2_O_2_ (2%) and acetic acid (0.1%)). All treatments significantly reduced mesophilic aerobic bacteria (1.29–4.08 log CFU/g), except alcohol (PK, RH) and sodium hypochlorite (PK). Reductions in enterobacteria (*p* < 0.05) ranged from 1.78 to 3.55 log CFU/g in RH; in PK, only the hot water + 2 chemical agents or 8% H_2_O_2_ treatments achieved significant reductions. The most effective treatment was hot water with H_2_O_2_ and acetic acid, which reduced bacteria and improved germination.

## 1. Introduction

Quinoa is an ancient grain native to the Andes that, due to its valuable nutritional contribution, functional characteristics, and agronomic versatility, is considered a strategic crop with the potential to contribute to food security [1,2,3]. In recent years, there has been an observed increase in the consumption of sprouts and quinoa, with the latter available in various forms, including whole grains, flours, bakery products, gluten-free products, and cereal bars, among others [2,4,5]. Sprouted vegetable seeds, whether in the form of whole grains, cut or crushed grains, or flour [6], have been favorably included in the diets of various population groups. Therefore, quinoa sprouts and their derivatives represent a promising alternative for consumers, aligning with the increasing interest in both healthy and sustainable nutrition. These market trends are expected to continue, with projections estimating that the global sprouted products market will reach a value of USD 12.84 billion by 2032 [7]. Within this context, quinoa represents a high-potential crop, with the global quinoa seed market valued at approximately USD 1 billion in 2024 and projected to grow to USD 2.07 billion by 2032 [8]. In the specific case of sprouted quinoa, products known as “sprouted quinoa crisps” have begun to gain market presence, with an estimated value of USD 344 million in 2024 and a projected increase to USD 732 million by 2033 (CAGR: 9.1%) [9].

Positive changes in nutritional composition, functional properties, biological characteristics, and even sensory attributes combined with the ease of implementation, minimal disruption, and a focus on environmental sustainability position germination as a viable alternative for grain processing and as an innovative approach to its consumption [6,10,11]. Germination involves soaking grains, followed by incubation, to produce sprouts—a process that triggers internal changes driven by humidity, temperature, and time. However, these same conditions and the availability of nutrients could potentially have a negative impact by favoring an increase in the initial microbial load, thereby affecting the microbiological quality of germinated products [4,6,12]. Quinoa grains, like other grains, carry an initial microbial load due to contamination during the growth, harvesting, and storage stages. In the case of quinoa, this may include total mesophilic bacteria, molds, and yeasts, with microbial loads varying approximately between 5.20 and 5.80 log CFU/g, 2.20 and 2.70 log CFU/g, 1.70 and 2.70 log CFU/g, respectively, and a single reported value of 3.80 log CFU/g for enterobacteria [4,13].

Many foodborne illnesses have been reported as a result of outbreaks associated with sprouts, and only a few disinfection treatments have been successful, especially when considering the need to preserve the viability of the embryo, minimize environmental impact, and avoid risks to human health [14,15]. Since 1988, more than 60 outbreaks have been associated with sprouts—primarily due to *Salmonella* and *Escherichia coli* O157:H7—even when following recommended handling practices [12,16]. This highlights the urgent need for effective, scalable, and safe disinfection strategies for seeds.

Intrinsic characteristics such as low water activity in grains further complicate disinfection, as they may increase bacterial resistance to heat and other decontamination methods. Therefore, seed disinfection is a crucial step in the germination process for ensuring public health. Conventional antimicrobial treatments applied to food at an industrial level, including the use of heat (hot water, steam) and chemical agents such as chlorinated compounds (sodium hypochlorite, chlorine dioxide), organic acids (peracetic acid), and quaternary ammonium compounds, have shown limited effectiveness for sprouts, requiring high concentrations that do not align with good manufacturing and safety practices [4,12,17]. Unconventional methods, such as plasma, ultrasound, gamma irradiation, ultraviolet irradiation, pulsed light, high pressure, microwave, and ozone treatment, among others, have been evaluated for grain disinfection. However, these treatments are not always effective, considering that disinfection processes can vary depending on factors such as seed type and germination conditions [4]. Furthermore, despite showing promising results at laboratory scale, many of these strategies still face limitations related to cost, infrastructure, and lack of long-term validation. The absence of standardized protocols also hinders their reliable application in real food systems. Therefore, it is necessary to continue investigating integrated disinfection strategies that ensure microbiological safety without compromising the functional properties of germinated grains like quinoa.

In this context, the USA Food and Drug Administration [12] recommends the use of scientifically validated disinfection methods (based on scientific information or data) that comply with relevant approvals and legislation. Furthermore, it emphasizes that physical and combined treatments have proven to be the most effective [18,19,20].

This study aimed to evaluate the effect of common disinfectants and combined treatments on the number of viable cells (CFU/g) of mesophilic aerobic bacteria and enterobacteria in germinated quinoa flours of Rosada de Huancayo (RH) and Pasankalla (PK) varieties from Peru. Although previous studies have evaluated disinfection procedures in quinoa grains from other origins [21], to the best of our knowledge, no study has assessed the impact of combined disinfection treatments and emergent technologies, such as ultrasound, on the microbiological quality and germination efficacy of Peruvian quinoa grains. The results could serve as a valuable reference for improving the quality and safety of quinoa sprouts, particularly when used in flour production.

## 2. Materials and Methods

### 2.1. Seed Samples

Two varieties of quinoa, Rosada de Huancayo (RH) and Pasankalla (PK), from Junín, Peru, were provided by the Universidad Nacional Agraria La Molina for quinoa sprout production. The quinoa grains were manually cleaned to remove impurities, such as leaves, stem fragments, and other foreign materials, and then stored at 4 °C for further use.

### 2.2. Preparation of Chemical Solutions

Five chemical solutions were prepared to evaluate their effectiveness in inactivating mesophilic aerobic bacteria and enterobacteria potentially present in quinoa grains. The solutions included sodium hypochlorite (NaOCl) at 200 ppm (*w*/*v*) (commercial bleach containing 4.5% sodium hypochlorite), acetic acid at 4% (*v*/*v*) (≥99.8%, Fluka, Seelze, Germany), ethanol at 70% (*v*/*v*) (96%, Bchem, Santo Antão do Tojal, Portugal), hydrogen peroxide (H_2_O_2_) at 8% (*v*/*v*) (30%, Carlo Erba, Val de Reuil, France), and a combination of hydrogen peroxide at 2% (*v*/*v*) and acetic acid at 0.1% (*v*/*v*). All solutions were prepared under aseptic conditions using sterilized water as the solvent.

### 2.3. Disinfection Procedures

Prior to carrying out the disinfection procedures, quinoa grains from each variety were weighed and washed with tap water to remove adhered soil, surface residues, and to perform desaponification. Seven disinfection procedures were evaluated, including chemical agents (sodium hypochlorite, acetic acid, ethanol, and hydrogen peroxide) and combined treatments (sodium hypochlorite with ultrasound; hot water with hydrogen peroxide and acetic acid). After washing the grains, excess water was drained, and the grains were subsequently dispersed in the respective disinfectant solutions. Disinfection with 200 ppm sodium hypochlorite (200 ppm SH) was carried out by submerging the quinoa grains for 30 min. The same procedure was applied for the 4% acetic acid (4% AA) solution, 70% ethanol (70% EtOH), and 8% hydrogen peroxide (8% H_2_O_2_), with immersion times of 15, 10, and 20 min, respectively. All procedures were conducted at room temperature (approximately 20 °C) using a sample-to-solution ratio of 1:5 (*w*/*v*).

The ultrasound treatment was carried out in an ultrasound bath (SONOREX SUPER RK 100 H, BANDELIN, Berlin, Germany) at a frequency of 35 kHz. For this, 200 g of previously weighed and washed quinoa was submerged in 2 L of 200 ppm sodium hypochlorite for 15 min (US15) and 30 min (US30), with a controlled temperature of 20 ± 2 °C. The sodium hypochlorite concentration was based on previous studies with alfalfa, buckwheat, and wheat sprouts while ensuring organic and safe production [22,23,24]. Exposure times were chosen from a combined ultrasound and chemical agent treatment that achieved the highest microbial reductions in soybean sprouts [25].

The hot water treatment, combined with hydrogen peroxide and acetic acid (HW + H_2_O_2_ + AA), was performed according to the method described by Wang et al. (2020) [26], with some modifications. Specifically, 30 g of each variety of quinoa was placed in bottles containing 150 mL of sterile water that had been previously heated to 50 °C. The bottles were placed in a water bath for 10 min and shaken every minute. Afterward, the samples were filtered under aseptic conditions and rinsed with sterile distilled water at room temperature to reduce seed temperature before chemical disinfection. The cooled samples were then immersed for 10 min in a solution of hydrogen peroxide (2% *v*/*v*) combined with acetic acid (0.1% *v*/*v*), followed by a second filtration process.

In all cases, following the completed disinfection procedure, the samples were rinsed with sterile distilled water to remove any residual disinfectant. This rinsing step consisted of two consecutive washes with sterile distilled water, each lasting 30 s. A control group without any disinfection treatment was included. All procedures, including the control, were performed in triplicate.

### 2.4. Germination Process and Sprouted Quinoa Flour

The germination of both quinoa varieties was conducted using the methodology developed by Paucar-Menacho et al. (2018) [27] with some modifications. Quinoa grains, both disinfected and non-disinfected (control), were soaked in sterile water (1:5, quinoa– water) for six hours at room temperature, with manual agitation every 30 min. After filtering the samples, they were placed in an incubator (Memmert, UFP 700, Schwabach, Germany) at 20 °C for 72 h, with periodic water spraying every six hours to maintain a relative humidity between 85 and 90%. The germination was carried out in darkness, and the incubation chamber operated under standard ventilation conditions. Once germination was completed, the samples were dried at 40 °C for 8 h (Memmert, Model 100–800, Germany), ground, and sieved (500 µm). The germinated quinoa flours were packaged in plastic bags and stored at 4 °C until analysis.

### 2.5. Microbiological Analysis

Microbiological analysis was conducted on the germinated quinoa flours. Under aseptic conditions, suspensions were prepared from 10 g of flour and 90 mL of 0.1% peptone (Himedia, Thane, India) solution, which were homogenized (Seward, Stomacher 400, Worthing, UK) for 1 min. Successive dilutions were made, and 1 mL of each suspension was poured onto a sterile Petri dish, to which 12 to 15 mL of the culture medium was added. Both were carefully mixed by rotating the Petri dish. Once the culture media solidified, the plates were inverted and placed in the incubator. For the enumeration of viable mesophilic aerobic bacteria (ISO 4833-1 [28]), plate count agar (PCA, Biokar Diagnostics, Allonne, France) was used, and the plates were incubated at 30 ± 1 °C for 72 ± 3 h. For the enumeration of enterobacteria (ISO 21528-2 [29]), violet red bile glucose agar (VRBG agar, VWR, Radnor, PA, USA) was used, and the plates were incubated at 37 °C for 24 ± 2 h. The culture media were prepared following the instructions from the manufacturer. Plate count agar (PCA) contained tryptone (5 g/L), glucose (1 g/L), yeast extract (2.5 g/L), and agar (12 g/L) and was sterilized by autoclaving at 121 °C for 15 min. Violet red bile glucose (VRBG) agar contained peptone (7 g/L), yeast extract (3 g/L), sodium chloride (5 g/L), glucose (10 g/L), bile salts (1.5 g/L), neutral red (0.03 g/L), crystal violet (0.002 g/L), and agar (13.0 g/L) and was sterilized by boiling for 1 min.

Colony counts were performed using a colony counter (Colony counter 570, Suntex, Taipei, Taiwan). For mesophilic bacteria, colonies on plates with fewer than 300 CFU were counted. For enterobacteria, plates with fewer than 150 colonies were selected, featuring pink to red colonies, with or without characteristic precipitation halos. Results were expressed as colony-forming units per gram of sample (log 10 CFU g^−1^ of sample).

### 2.6. Germination Power

The impact of disinfection treatments on the germination power of quinoa grains was assessed. Samples of 100 quinoa grains, previously washed and disinfected as described in previous sections, were placed on 150 mm diameter Petri dishes containing filter paper moistened with 4 mL of distilled water. The plates were incubated in darkness at 24 ± 2 °C, and the number of germinated grains was evaluated every 12 h for a maximum period of 72 h to determine the germination percentage [30].

### 2.7. Statistical Analysis

All experiments were conducted in triplicate, and results were expressed as mean ± standard deviation (SD). Data normality and homogeneity of variance were assessed using the Shapiro–Wilk and Levene’s tests, respectively. Significant differences between treatments were determined using one-way analysis of variance (ANOVA), followed by Tukey’s post hoc test to identify pairwise differences among means. The significance level was set at *p* < 0.05. Statistical analyses were performed using IBM SPSS Statistics 29.0 (IBM Corp., Armonk, NY, USA).

## 3. Results and Discussion

### 3.1. Effect of Disinfection on Mesophilic and Enterobacterial Counts

The use of chemical products and combined techniques for disinfecting food and food facilities has demonstrated high efficacy in most cases; however, variability in results has been observed, particularly with fresh products (including sprouts), making outcomes less promising [4,18,31]. Various disinfectants and disinfection techniques are routinely employed in the food industry. However, selecting the right chemical and/or method is crucial to achieving the desired effect. The choice depends on factors such as the type of microorganism, the lethality (efficacy) of the disinfectant against the specific microorganism, the impact on food quality and safety, risks to operators and the environment, and costs, among others [31,32,33].

Regarding the use of physical treatments such as irradiation (gamma or electron beam) and gaseous ozone for seed disinfection, irradiation technologies have proven to be highly effective in reducing pathogens in seeds such as tomato, cantaloupe, and lettuce, reaching or even exceeding reductions of 5 log CFU/g. However, these techniques require high doses, generally between 2 and 8 kGy depending on the type of pathogenic microorganism and seed, which can compromise seed viability—especially radicle development—if inappropriate doses are applied. Moreover, these treatments demand specialized infrastructure, trained personnel, and strict safety protocols, which limits their applicability outside industrial environments [34,35]. In the specific case of quinoa, recent research on electron beam irradiation (EBI) has shown that doses up to 8 kGy can effectively control microbial growth during storage while preserving seed quality. In particular, EBI was reported to reduce lipase activity and free fatty acid accumulation, while maintaining saponin content, decreasing phytic acid levels, and even improving certain phytochemical attributes such as total phenolic content [36].

On the other hand, ozone has been shown to be a potent disinfectant, capable of achieving microbial reductions of around 3–4 log CFU/g without leaving chemical residues and, in general, without affecting germination capacity. For example, de Alencar et al. (2021) [37] observed that the application of gaseous ozone at 885 ppm for 120 min did not negatively affect the germination or vigor of quinoa seeds. Similarly, Trinetta et al. (2011) [34] reported that gaseous ozone treatment achieved reductions of up to 4 log CFU/g of *Salmonella* and *E. coli* O157:H7 in tomato, melon, and lettuce seeds, without observing significant effects on their germination ability. Despite these promising results, ozone use carries risks associated with its toxicity as an inhalable gas. It requires on-site generation and carefully controlled treatment conditions to avoid adverse effects on both the seeds and the working environment [38]. Although more accessible than irradiation, it remains a limited option for household or small-scale applications.

**Figure 1:**
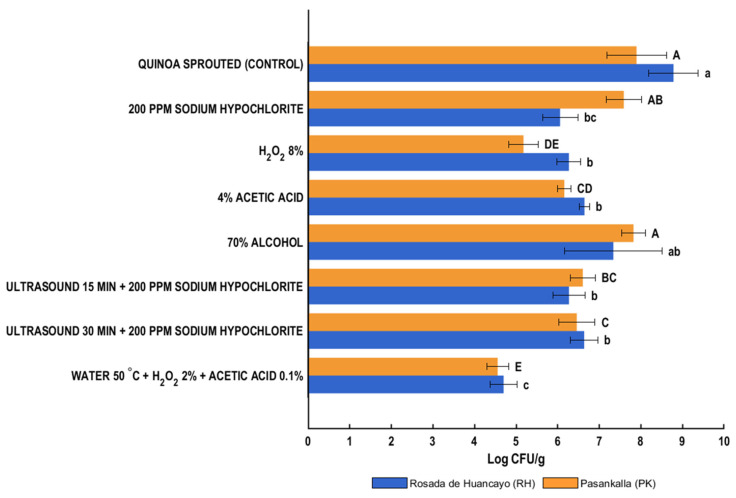
Viable counts of mesophilic aerobic bacteria (log CFU/g) in germinated quinoa flours of Pasankalla (PK) and Rosada de Huancayo (RH) varieties after different disinfection treatments. Values are mean ± SD (n = 3). Bars labeled with different letters are significantly different (Tukey’s test, *p* < 0.05). Uppercase letters: Pasankalla (PK); lowercase letters: Rosada de Huancayo (RH).

Figure 1 shows the effect of seven disinfection treatments on the mesophilic aerobic bacteria population in grains of two quinoa varieties. In all cases, the disinfection procedures reduced the microbial load compared to the untreated samples (control). The reductions ranged from 1.45 to 4.08 log CFU/g for RH quinoa flour and from 0.07 to 3.34 log CFU/g for PK quinoa flour.

For the RH variety, microbial counts ranged from 8.78 log CFU/g in the control sample to 4.70 log CFU/g in the sample treated with hot water + H_2_O_2_ + acetic acid, the most effective combination (4.08 log cycles reduction in mesophilic counts). Among the chemical disinfectants, 70% ethanol showed the highest microbial load (7.34 log CFU/g) and did not differ significantly from the control, indicating low efficacy. In contrast, 200 ppm sodium hypochlorite reduced the microbial population to 6.06 log CFU/g, showing a statistically significant reduction (2.73 log cycles reduction) and being the most effective chemical disinfectant tested. For the PK variety, microbial counts ranged from 7.90 log CFU/g in the control sample to 4.56 log CFU/g in the treatment with hot water + H_2_O_2_ + acetic acid, which was again the most effective (3.34 log cycles reduction in mesophilic counts). Within the chemical disinfectants, 70% ethanol showed the highest microbial load (7.82 log CFU/g) and did not differ significantly from the control, confirming its limited antimicrobial effect. In contrast, 8% hydrogen peroxide reduced the microbial population to 5.18 log CFU/g, representing the most effective chemical treatment with a statistically significant reduction (2.72 log cycles reduction).

**Figure 2:**
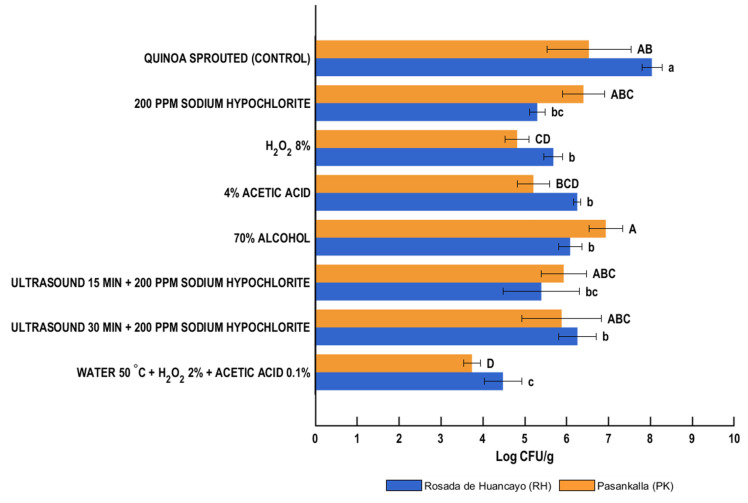
Viable counts of enterobacteria (log CFU/g) in germinated quinoa flours of Pasankalla (PK) and Rosada de Huancayo (RH) varieties after different disinfection treatments. Values are mean ± SD (n = 3). Bars labeled with different letters are significantly different (Tukey’s test, *p* < 0.05). Uppercase letters: Pasankalla (PK); lowercase letters: Rosada de Huancayo (RH).

A similar effect was observed in the enterobacteria population, with reductions ranging from 1.78 to 3.56 log CFU/g for the RH variety, and from 0.13 to 2.79 log CFU/g for the PK variety (Figure 2), except for the treatment where 70% alcohol was applied to the PK variety. In addition, in the absence of any disinfection treatment (control group), the RH variety exhibited the highest counts of aerobic mesophilic bacteria (9.41 × 10^8^ CFU/g) and enterobacteria (1.21 × 10^8^ CFU/g), in comparison to the PK variety, which displayed counts of 1.41 × 10^8^ CFU/g and 1.68 × 10^7^ CFU/g, respectively.

For the RH variety, microbial counts of enterobacteria ranged from 8.04 log CFU/g in the control sample to 4.48 log CFU/g in the treatment with hot water + H_2_O_2_ + acetic acid (*p* < 0.05) (3.56 log cycles reduction in enterobacteria). Among the chemical disinfectants, 4% acetic acid showed the highest microbial count (6.25 log CFU/g), although it differed significantly from the control. In contrast, 200 ppm sodium hypochlorite was the most effective chemical treatment, reducing the microbial load to 5.30 log CFU/g, with statistically significant differences (2.74 log cycles reduction). For the PK variety, enterobacteria counts ranged from 6.53 log CFU/g in the control to 3.74 log CFU/g in the sample treated with hot water + H_2_O_2_ + acetic acid, representing the greatest reduction (*p* < 0.05) (2.79 log cycles reduction in enterobacteria). The 70% ethanol treatment showed the least effective reduction (6.94 log CFU/g), with no significant difference from the control. On the other hand, 8% hydrogen peroxide again stood out as the most effective chemical agent, reducing enterobacteria to 4.82 log CFU/g (1.71 log cycles reduction).

#### 3.1.1. Chemical Treatments

In the germinated quinoa flour of the PK variety, the least reductions in mesophilic aerobic bacteria counts were observed when common disinfectants such as alcohol (70%) and sodium hypochlorite (200 ppm) were used. The reductions were 0.31 and 0.07 log CFU/g (*p* > 0.05), respectively, compared to the control. In the germinated quinoa flour of the RH variety, the effect of the alcoholic solution was more pronounced (with a reduction of 1.45 log CFU/g), although not sufficient to be considered significant. This treatment produced the least reduction in the bacterial population. In this variety, the behavior of sodium hypochlorite differed, achieving the second-highest reduction in viable cell numbers (2.73 log CFU/g, *p* < 0.05), second only to the treatment using hot water (50 °C) + hydrogen peroxide (2%) + acetic acid (0.1%).

Similarly to mesophilic bacteria, in enterobacteria, sodium hypochlorite (200 ppm) and alcohol (70%) were the disinfectants with the least positive effect on the PK variety, with a reduction of only 0.13 log CFU/g when the former was applied, while the latter even showed a slight increase in the population. In the RH variety, sodium hypochlorite again produced the second-highest reduction in viable cell numbers (2.74 log CFU/g, *p* < 0.05), but it did not show significant differences compared to other disinfection treatments. Alcohol (70%) caused a reduction of 1.95 log CFU/g in viable cell numbers, corresponding to a 24.3% decrease.

Within the group of chemical disinfectants, sodium hypochlorite is one of the most common and widely used in the food industry. Its low cost, easy accessibility, and demonstrated ability to reduce microbial load make it a favored choice [31,33,39]. The effectiveness of its action is related to its chemical forms in solution (Cl_2_, HOCl, and the hypochlorite anion, OCl^−^), which, in turn, depend on concentration, solution pH, and exposure time [33,39]. The use of sodium hypochlorite (NaOCl) has been evaluated for the reduction of *E. coli*, *L. monocytogenes*, *S. typhimurium*, and *S. enterica* in minimally processed foods (fruits and vegetables), yielding values that are not always satisfactory (0.7 to 5.5 log CFU/g) for products such as spinach, lettuce, Swiss chard, cabbage, and tomatoes [33]. In the disinfection of alfalfa seeds, concentrations of 200 to 2000 ppm of NaOCl had a minimal effect on the population of *Salmonella* (reductions of 0.33 and 0.72 log CFU/g in viable cell numbers, respectively) [17]. For *E. coli* O157:H7, concentrations exceeding 2000 ppm were required to achieve a significant reduction [22]. In sprouts, disinfection with chlorine-based aqueous solutions, such as acidified electrolyzed water (AEW) or slightly acidic electrolyzed water (SAEW), has achieved microbial reductions ranging from 0.98 to 4.24 log CFU/g against *E. coli* O157:H7, depending on concentration and exposure time [4]. For example, when sodium hypochlorite solutions (containing approximately 200 mg/L available chlorine for 10 min) were used to disinfect buckwheat, the reduction in total bacterial count reached 1.29 log CFU/g [23]. When wheat seeds were disinfected with NaOCl (100, 200, and 400 ppm) and then germinated for a period of 9 days, the total population of aerobic mesophilic bacteria, total coliforms, and yeast and molds showed a reduction from 0.3 to 1.46, 0.03 to 0.84, and 0.29 to 1.93 log CFU/mL in viable cell numbers, respectively, compared to the non-disinfected sample (control). In all cases, the greatest reductions were observed when the highest concentration, 400 ppm, was applied [24]. Both the FDA [12] and CFIA [40] recognize sodium hypochlorite as a viable antimicrobial treatment for seeds in sprout production but indicate that achieving at least a 3 log CFU/g reduction in pathogenic bacteria requires concentrations of 19,000 ppm or 2000 ppm, respectively. Such concentrations would conflict with organic production standards and could pose significant environmental and health risks to operators [18,26,41]. The results obtained in this study using concentrations 10 times lower (200 ppm) than that recommended by the CFIA [40] and approximately 95 times lower than that indicated by the FDA [12], especially in the RH variety, reached values similar to those achieved in other studies and were even close to the minimum indicated by these organizations.

On the other hand, the results obtained with the 70% alcohol solution reflect its low effectiveness for quinoa grain disinfection. A significant reduction of 1.95 log CFU/g in the enterobacteria population was achieved only in the RH variety. Few studies have evaluated alcohol as a direct disinfectant for food. In the case of grains or seeds, studies have focused on the surface disinfection of seeds to promote the growth of healthy seedlings and the production of edible sprouts, although these studies are limited [42,43,44]. In sprout production, when alfalfa seeds were exposed to an 80% ethanol solution for 30 s, the *Salmonella* content was reduced by up to 1000 times [45]. Concentrations of 30% and 70% alcohol achieved significant reductions in *E. coli* when the immersion time was 3 and 10 min, with a greater effect in the latter case [22]. Similar behaviors were observed in a combined treatment with dry heat + 50% ethanol, where the population of *E. coli* O157:H7 was reduced to non-detectable levels in mung bean, radish, broccoli, and alfalfa seeds. However, this did not imply complete inactivation of the microorganism [46].

Organic acids, including acetic acid, are widely utilized in the food industry due to their antimicrobial properties. Their effect on microbial cells, by modifying pH and affecting intracellular activities, leads to cell death, thereby contributing to food safety. However, their efficacy is variable and depends on the morphological and structural properties of microorganisms [47]. Hydrogen peroxide (H_2_O_2_) is a potent oxidizing agent that reduces microbial load by generating multiple oxidative species, causing damage to intracellular compounds of microorganisms, including DNA and proteins [41]. These chemical agents are considered environmentally friendly and safe for use in food. However, challenges such as quality issues caused by acetic acid and risks in handling hydrogen peroxide make their use complicated. Therefore, it is necessary to evaluate the advantages and challenges of using these substances for grain disinfection and subsequent sprout production [26,41,47]. In the quinoa grains, solutions of acetic acid (4%) and hydrogen peroxide (8%) significantly reduced the population of mesophilic bacteria in germinated quinoa flours (*p* < 0.05) compared to the control. The effect of 4% acetic acid was greater in the RH variety compared to PK (24.4% vs. 21.9% reduction, respectively). In comparison, 8% hydrogen peroxide achieved a greater reduction in the PK variety (34.4% vs. 28.6%, for PK and RH, respectively). As shown in Figure 1, hydrogen peroxide had a greater bactericidal effect than acetic acid in both varieties, achieving reductions of 2.51 (RH) and 2.72 (PK) log CFU/g in viable cell numbers. For the enterobacteria population, 4% acetic acid and 8% hydrogen peroxide showed a behavior similar to that observed for mesophilic bacteria. Specifically, 8% hydrogen peroxide had a greater impact on the microbial population in both varieties, achieving significant reductions of 2.35 and 1.71 log CFU/g in viable cell numbers in the RH and PK varieties, respectively. Acetic acid reduced the enterobacteria population by 1.78 log CFU/g (RH) and 1.32 log CFU/g (PK), with the latter being non-significant (*p* > 0.05) with respect to the control (Figure 2). Studies on alfalfa and mung bean sprouts evaluated the efficacy of 2% and 5% acetic acid solutions. In both cases, they achieved the elimination of *Salmonella* (reductions between 7 and 8 log CFU/g) when immersed for a minimum of 24 and 4 h (alfalfa) and 48 and 16 h (mung bean), respectively. In mung beans, treatments with 5% acetic acid for 3 h reduced the concentration of these microorganisms but did not achieve complete elimination [48,49]. In studies conducted on alfalfa seeds, reductions of 0.39 log CFU/g and 1.74 log CFU/g in the *Salmonella* populations were achieved when 2% and 5% acetic acid solutions were used for 10 min, respectively [17]. In the case of *E. coli* O157:H7, a 5% acetic acid solution (10 min, 42 °C) resulted in a 6.3 log reduction in CFU/g. Attempts to improve efficacy through alternative application methods have been explored. For example, gaseous acetic acid at 8.7% (*v*/*v*) applied to the disinfection of radish and alfalfa seeds managed to eliminate the *E. coli* O157:H7 population after 48 h; however, the *Salmonella* population was only partially reduced [50]. In quinoa grains of both evaluated varieties (RH and PK), the antimicrobial effect of acetic acid was similar to or even exceeded the reductions reported for *Salmonella*. Differences with other studies may be attributed to variations in concentration, time, temperature, and the reference microorganism used.

Several investigations on alfalfa seeds have evaluated the effect of hydrogen peroxide as a disinfectant [17,22,26,30,45]. For instance, a solution of 6% hydrogen peroxide achieved a reduction greater than 3 log CFU/g times in the *Salmonella* population [45]. While 8% hydrogen peroxide solutions produced reductions of 3.22, 4.27, 3.69, 2.77, and greater than 3.92 log CFU/g in viable cell numbers, with the last three being observed in non-scarified, scarified, and polished seeds [17,26,30]. In addition, significant reductions in the *E. coli* population on alfalfa seeds were reported when a 0.2% solution was used, while concentrations of 1% or higher achieved complete elimination [22]. Similar behaviors were observed in non-scarified, scarified, and polished seeds, where the application of an 8% hydrogen peroxide solution reduced the *E. coli* population by 3.60, 2.30, and 3.56 log CFU/g, respectively [30]. In radish seeds, an 8% hydrogen peroxide solution (10 min, room temperature) reduced the population of *E. coli* O157:H7, *Salmonella enterica*, and *Listeria monocytogenes* by 1.88, 2.13, and 1.50 log CFU/g, respectively. The same solution applied to alfalfa achieved greater reductions (4.52, 4.27, and 4.40 log CFU/g, respectively) in viable cell numbers [26]. In the quinoa grains (RH and PK varieties), when the same concentration and time were applied, the reduction obtained was lower than that achieved in alfalfa and higher than that obtained in radish seeds, highlighting that the bactericidal effect is highly dependent on the type of raw material and the microorganism to be eliminated. In another study, solutions of 3% and 6% hydrogen peroxide reduced the population of total aerobic mesophilic bacteria (0.09 and 0.95 log CFU/g), total coliforms (0.23 and 0.28 log CFU/g), and yeast and molds (0.67 and 1.03 log CFU/g) in disinfected and germinated wheat seeds after nine days [24]. The concentration of hydrogen peroxide used in the quinoa grains was within the range where the greatest effect of this chemical agent has been observed. While the reduction achieved was not as high as in most studies using *Salmonella* and *E. coli* as reference microorganisms, it was higher than that obtained for wheat when the population of total aerobic mesophilic bacteria and total coliforms was evaluated. Additionally, the FDA [12] and the CFIA [40] consider hydrogen peroxide a chemical agent that can be applied to reduce the microbial load in sprouts, recommending concentrations between 6% and 10%. Regarding acetic acid, the FDA considers the use of gaseous acetic acid (8.7% *v*/*v*) an antimicrobial treatment on a large scale [12].

#### 3.1.2. Combined Treatments

In this study, the efficacy of combined methods was evaluated, specifically ultrasound (35 kHz) with sodium hypochlorite (200 ppm) and hot water (50 °C) with hydrogen peroxide (2%) and acetic acid (0.1%), on the RH and PK quinoa varieties. The two ultrasound-assisted combined treatments (ultrasound with sodium hypochlorite, 15 and 30 min) achieved significant reductions (*p* < 0.05) compared to the control, except for the population of enterobacteria in the PK variety. However, these reductions were not as high as those observed with the hot water with H_2_O_2_ and acetic acid treatment or when sodium hypochlorite, hydrogen peroxide, and acetic acid solutions were used individually. This aligns with observations from some authors [18,31,51] indicating that combined treatments may not be as effective in some cases. According to Mir et al. (2021) [4] and Gilbert et al. (2023) [51], ultrasound is not highly effective against certain bacteria and fungi, and its implementation on an industrial level has not yet been widely adopted. Evaluations conducted on seeds and sprouts revealed reductions between 0.35 and 4.05 log CFU/g for pathogenic bacteria (*Salmonella*, *E. coli*, *E. coli* 0157:H7), 0.6 and 6.41 log CFU/g for total aerobic bacteria, and 0.52 and 1.42 log CFU/g for total coliforms after ultrasound application. Frequencies ranging from 20 to 40.5 kHz and exposure times of 1 to 30 min were employed. When ultrasound was combined with other technologies, reductions in pathogenic bacteria were reported to be 0.57–6.00 log CFU/g, including *Salmonella*, *E. coli*, *E. coli* 0157:H7, and *Listeria monocytogenes* [52]. In the quinoa grains, ultrasound achieved a reduction in mesophilic population of 2.51 (RH) and 1.29 (PK) log CFU/g with a 15 min immersion, and 2.15 (RH) and 1.44 (PK) log CFU/g with a 30 min immersion. For enterobacteria, reductions with a 15 min application were 2.64 (RH) and 0.60 (PK) log CFU/g, and with a 30 min application, the population decreased by 1.78 (RH) and 0.65 (PK) log CFU/g. The application time did not influence the reductions in microbial population for both types of microorganisms (Figure 1 and Figure 2), but a lesser effect was observed on the PK variety. Although some studies report reductions equal to or greater than 6 log CFU/g when ultrasound was used individually or in combination [25], most investigations on seeds and sprouts indicate reductions not exceeding 2 log CFU/g [52], consistent with the results obtained in the quinoa grains evaluated (RH and PK varieties).

Among all the treatments evaluated, including those involving ultrasound, the one that exhibited the highest reduction (*p* < 0.05) in both quinoa varieties was the treatment involving heat (hot water), followed by the simultaneous application of two chemical agents (H_2_O_2_ and acetic acid). This was observed for both mesophilic bacteria and enterobacteria populations. The RH variety showed the most significant reduction in the mesophilic bacteria population (4.08 log CFU/g, 46.5% reduction), while the PK variety exhibited the smallest reduction in the enterobacteria population (2.79 log CFU/g, 42.7% reduction), in terms of log CFU/g. However, when assessing percentage reductions (comparing the initial and reduced populations), the least reduction was observed in the mesophilic bacterial population of the PK variety (3.34 log CFU/g, a 42.3% reduction). The use of hot water and dry heat has been studied as a disinfection method for germinated products [18,53]. Reductions between 4.19 and 6.9 log CFU/g in the microbial pathogen population were observed when seeds (alfalfa, radish, and mung bean) and/or sprouts (alfalfa, radish, mung bean, and sunflower) were immersed in hot water [4]. The elimination of *Salmonella* was achieved in alfalfa and mung beans when temperatures of 70 °C for 10 s and 20 s, respectively, were applied [48]. Heat’s destructive impact on the cell membrane, enzymes, and proteins seems efficient for microbial destruction. However, it is crucial to consider the temperatures and times employed to avoid adverse effects on texture, color, germination percentage, and, of course, nutritional quality [4,53]. In this regard, the use of combined treatments could enhance the effectiveness of physical and chemical agents, mitigating undesirable effects. In studies conducted on mung bean seeds for sprout production, combined treatments (T1: 85 °C water, 10 s + 2000 ppm chlorine, 2 h; T2: 20,000 ppm calcium chlorite, 20 min + 2000 ppm chlorine, 2 h) achieved a greater reduction in the population of *E. coli* O157:H7, *Salmonella*, and nonpathogenic *E. coli* compared to simple methods (T3: 85 °C water, 10 s; T4: 20,000 ppm calcium chlorite). For *E. coli* O157: H7, the population reduced by 0.71 (T1) and 0.41 (T2) log CFU/g, nonpathogenic *E. coli* experienced a reduction of 0.91 (T1 and T2) log CFU/g, and *Salmonella* only had a reduction of 0.51 log CFU/g with the chemical + chemical treatment (T2) [54]. In alfalfa seeds, the use of dry heat (60, 70, or 80 °C) followed by the application of H_2_O_2_ (2%) resulted in a reduction between 1.66 and 3.60 log CFU/g in the population of *Salmonella typhimurium*. When only dry heat was applied, the reduction ranged from 0.26 to 2.76 log CFU/g [55], representing a decrease in effectiveness of approximately 23.33% to 84.34% compared to the combined treatment. The same treatment applied to the evaluated quinoa seeds (hot water: 50 °C × 10 min combined with H_2_O_2_ (2%) and acetic acid (0.1%): 10 min) in alfalfa and radish seeds resulted in reductions of 4.60 and 1.74 log CFU/g for *E. coli* O157:H7, 5.02 and 1.18 log CFU/g for *Salmonella enterica*, and 4.59 and 2.04 log CFU/g for *Listeria monocytogenes* [26], respectively. The outcomes achieved in this study in both quinoa varieties fell within the values obtained for these two types of seeds. Apparently, the combined effect of heat, hydrogen peroxide, and acetic acid yields a greater bactericidal effect on mesophilic bacteria and enterobacteria present in the evaluated quinoa grain varieties, compared to when only one chemical agent or ultrasound is used.

When considering the practical application of these treatments, in terms of the microbiological characteristics of the flours, only the use of 8% hydrogen peroxide (PK variety) and hot water + H_2_O_2_ + acetic acid (both varieties) led to a reduction in the population of aerobic mesophilic bacteria to permissible levels (≤10^6^ CFU/g) according to technical guidelines such as those compiled by Moragas and Valcárcel (2021) [56]. These results indicate that the concentrations, time, and temperatures applied in these instances were sufficient to lower the microbial population to safe levels.

Supporting these observations, several studies have shown that hydrogen peroxide becomes significantly more effective as a disinfectant in acidic conditions. For example, a 3% H_2_O_2_ solution acidified with citric acid to a pH of 2.5 was reported to inactivate SARS-CoV-2 by more than 4 log units within just 5 min, while the same non-acidified solution achieved only a 1.1 log reduction in the same time [57]. Similarly, Raffellini et al. (2008) [58] studied the inactivation kinetics of Escherichia coli ATCC 35218 in hydrogen peroxide solutions with different concentrations (0–3%) and pH values (3.0–7.2), showing that both higher concentration and lower pH significantly increased its effectiveness. At low H_2_O_2_ concentrations, the influence of pH on the microbial inactivation rate was even more pronounced, clearly demonstrating a synergistic effect between the two factors. This behavior has been linked to the greater stability of hydrogen peroxide under acidic conditions. In this regard, Cords et al. (2005) [59] concluded that the bactericidal and sporicidal activity of H_2_O_2_ tends to be higher in acidic media, remains moderate at neutral pH, and decreases in alkaline environments. These findings support the importance of pH control as a key factor in the effectiveness of hydrogen peroxide-based disinfection treatments.

In the case of quinoa seeds from both varieties, the greater microbial load reduction could be attributed not only to the effect of the acidic medium on hydrogen peroxide, but also to the possible in situ formation of peracetic acid. This compound is generated by the reaction between acetic acid and hydrogen peroxide and is known for its high oxidative power and redox potential, which surpasses that of chlorine. Peracetic acid has been shown to exert strong antimicrobial activity against pathogenic bacteria due to its ability to oxidatively disrupt the bacterial cell membrane [60]. Additionally, studies on biofilms have reported that combining peracetic acid with heat can enhance microbial reduction. For instance, a 10 ppm peracetic acid solution reduced the Staphylococcus aureus biofilm population by 3.2 log CFU/cm^2^ at 25 °C over 10 min, while increasing the temperature to 50 °C led to a 5.1 log CFU/cm^2^ reduction [60].

Regarding the potential formation of undesired compounds, it is important to note that peracetic acid (PAA), which can be formed in situ from hydrogen peroxide and acetic acid, is approved for seed treatment in organic production systems according to USDA–NOP regulations, although no specific concentration limits are established for its application. This compound is considered safe, as its decomposition products—acetic acid, water, oxygen, and hydrogen peroxide—are harmless and do not pose significant health risks [61,62]. Sodium hypochlorite is widely used in the food industry as a disinfectant and is approved by the FDA for use as a sanitizing solution, including on surfaces in direct contact with food and for washing fruits and vegetables [61]. However, its application may generate undesired by-products such as chlorates, whose presence has been the subject of numerous studies due to their potential health effects. Nevertheless, studies conducted on vegetables such as lettuce have shown that proper rinsing after hypochlorite treatment can significantly reduce residual chlorate levels [63,64]. In this study, quinoa grains were rinsed with sterile water after each treatment, which reinforces the safety of the procedure by decreasing the likelihood of chemical residues. This same effect may apply to possible ethanol residues. Although ethanol is considered a GRAS (“Generally Recognized As Safe”) compound by the FDA [61], its use could lead to the formation of volatile by-products such as ethyl acetate. However, successive water rinses following disinfection likely helped eliminate or significantly reduce its presence in the treated grains.

It is important to note that, although all disinfection treatments were designed based on protocols supported by previous studies, there were differences in solution volume, sample mass, and exposure time among them. These conditions were not intentionally standardized, as each treatment requires specific parameters for its proper application and microbiological efficacy. This methodological heterogeneity may limit the direct comparability between treatments and represents a potential source of bias. However, this variability reflects the real-world operational use of each procedure, in which conditions must be adapted to the type of disinfectant and its mechanism of action. Therefore, the results should be interpreted considering that the aim of the study was to assess the practical effectiveness of treatments applicable in different production contexts, rather than to perform an absolute comparison under strictly uniform experimental conditions. This approach offers greater realism and relevance for designing adaptable protocols suitable for various settings (domestic, semi-industrial, or artisanal), although it entails certain limitations regarding the direct comparison of efficacy among all treatments evaluated. In addition, the microbiological evaluation in this study was based solely on the enumeration of colony-forming units (CFU), which allows the detection of culturable microorganisms but not other viable forms such as VBNC (viable but non-culturable) cells, nor does it detect potential sublethal damage to DNA or cell membranes. Similarly, the presence of bacterial spores or microorganisms in latent states was not specifically addressed. These limitations should be taken into account when interpreting the effectiveness of the treatments, especially in more demanding food safety contexts. In this regard, Li et al. (2014) [65] demonstrated that conventional culture methods can significantly underestimate microbial load when VBNC cells are present. Complementarily, Pazos-Rojas et al. (2023) [66] emphasized the growing importance of these viable but non-culturable forms in food environments and the need to apply molecular tools for their proper detection.

Techniques such as qPCR combined with the use of intercalating dyes (e.g., propidium monoazide or ethidium monoazide) allow the differentiation between viable and non-viable cells and could provide a more accurate assessment of the microbiological effectiveness of the treatments [67]. Therefore, in future studies, it would be advisable to complement this type of analysis with molecular approaches and selective recovery media for spore-forming bacteria in order to obtain a more comprehensive view of the microbiological impact of the applied methodologies.

### 3.2. Effect of Disinfection Procedures on Germination Power

Table 1 displays the germination power results for each quinoa variety and different disinfection treatments. Considering that germination was deemed achieved upon the emergence of radicles, the findings suggest that disinfection treatments had minimal impact on the germination power of the assessed grains. In fact, certain disinfection procedures demonstrated an increase in germination percentages with respect to the control.

**Figure 3:**
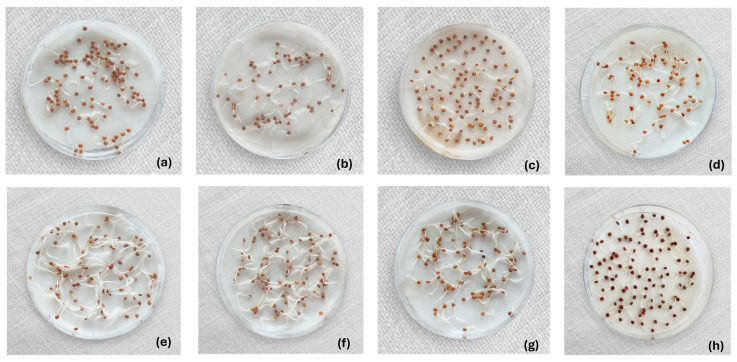
Visual comparison of Pasankalla (PK) quinoa grain germination after seven disinfection treatments: (**a**) 4% acetic acid; (**b**) 200 ppm sodium hypochlorite; (**c**) 70% alcohol (**d**) 8% hydrogen peroxide; (**e**) ultrasound 15 min + 200 ppm sodium hypochlorite; (**f**) ultrasound 30 min + 200 ppm sodium hypochlorite; (**g**) water 50 °C + 2% hydrogen peroxide + acetic acid 0.1%; (**h**) control.

In the PK variety, only the 70% alcohol treatment exhibited a reduction in the germinative capacity of approximately 10% of the grains (±5.51%) compared to the control (*p* < 0.05); however, all other treatments resulted in 100% germination. In this variety, regardless of the treatment applied, the generation of lengthy sprouts occurred, with dimensions varying based on the disinfection methodology employed (Figure 3). Thus, the combined treatments had the most positive impact on the radicles, while the treatments with acetic acid and alcohol were those in which the radicles were most affected.

**Figure 4:**
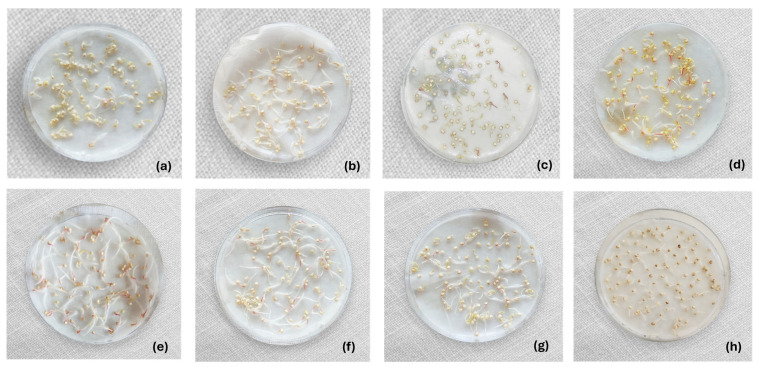
Visual comparison of Rosada de Huancayo (RH) quinoa grain germination after seven disinfection treatments: (**a**) 4% acetic acid; (**b**) 200 ppm sodium hypochlorite; (**c**) 70% alcohol (**d**) 8% hydrogen peroxide; (**e**) ultrasound 15 min + 200 ppm sodium hypochlorite; (**f**) ultrasound 30 min + 200 ppm sodium hypochlorite; (**g**) water 50 °C + 2% hydrogen peroxide + acetic acid 0.1%; (**h**) control.

In the RH variety, germination power saw reductions in treatments involving alcohol and acetic acid. Conversely, treatments utilizing sodium hypochlorite and 15 min ultrasound exposure resulted in an increased germination capacity compared to the control, achieving up to 100% germination of the grains. In this variety, the alcohol treatment notably impacted quinoa germination capacity, showing a maximum reduction of 9.5% compared to the control (*p* < 0.05). In comparison, the acetic acid treatment resulted in a maximum reduction of 5.05% (*p* < 0.05). Disinfection treatments in the RH variety exerted both positive and negative effects on radicle size, as observed in Figure 4. In this variety, the ultrasound treatments (15 and 30 min) and the sodium hypochlorite treatment individually had a positive impact on radicle size. In contrast, acetic acid and alcohol had the most negative effect, the latter being consistent with the PK variety. Similar outcomes were observed in three pea varieties, where decontamination using NaOCl (1 g/L), vinegar (350 mL), and ethanol (350 mL) yielded germination percentages ranging from 88.91% to 94.60%, 88.26% to 90.84%, and 87.23% to 92.96%, respectively. The application of ultrasound (40 kHz, 1 min, 25 °C) in these pea varieties resulted in higher germination percentages (96.30% to 98.18%) compared to treatments involving chemical disinfectants [68]. In contrast to the effects on quinoa grains, all disinfection treatments in peas enhanced the germination percentage compared to the control. In other investigations, the use of acetic acid and ethanol led to reductions in the germination percentage. For instance, in alfalfa seeds, ethanol (80%) reduced the germination percentage by 3% [45], and a 5% acetic acid solution applied for 10 min resulted in a germination percentage of only 46.7% [17].

The use of sodium hypochlorite and hydrogen peroxide has also been evaluated in terms of their germination power. In alfalfa seeds, the use of a sodium hypochlorite solution (200 ppm) did not affect seed germination compared to the control (93.3% vs. 93.5%) [17]. However, in another study conducted on alfalfa with the same concentration of sodium hypochlorite (200 ppm), the germination percentage was reduced by 3% [45]. Regarding the use of hydrogen peroxide, some studies suggest that it may reduce the germination percentage, depending on the concentration of the solution used. For example, in alfalfa seeds, a 6% solution resulted in a 4% reduction, while a concentration of 10% showed a slight increase in the germination percentage (1%) [45]. Nevertheless, this contradicts the expected outcomes, as numerous studies have reported that H_2_O_2_ could improve the germination percentage [69]. For instance, another study performed on alfalfa seeds showed that increasing the concentration of H_2_O_2_ from 0.2% to 2% led to a 2.8% and 3.2% increase in the germination percentage, respectively. When the solution had an 8% concentration, the increase was 2.9% [17]. In another study on alfalfa and radish seeds, the germination percentages were approximately 91% and 99%, respectively, when 8% H_2_O_2_ was applied for 10 min [26]. Regarding heat, some studies suggest that it could reduce the germination power of grains. However, this is dependent on the temperature and time applied, as well as the type of grain and other intrinsic factors [26,70,71,72]. Weiss and Hammes (2005) [71] reported that alfalfa seed is capable of tolerating temperatures between 55 and 64 °C for periods of 2 to 10 min. However, Jaquette et al. (1996) [72] demonstrated that temperatures exceeding 54 °C for 10 min result in a substantial reduction in the viability of these seeds. In RH quinoa, the combined effect of heat and two chemical agents (including H_2_O_2_) resulted in a slightly greater reduction in germination capacity compared to 8% H_2_O_2_ (98.00 ± 1.73% vs. 99.00 ± 0.00%, respectively), although this difference was not significant. In both quinoa varieties, applying a temperature of 50 °C for 10 min did not negatively affect the germination percentage; instead, it had a positive effect, increasing the number of germinated seeds compared to the control, though this increase did not exhibit statistical significance (RH: 98.00 ± 1.73% vs. 97.50 ± 3.02%, PK: 100.00 ± 0.00% vs. 99.67 ± 0.52%, respectively) (Table 1). This was also observed in alfalfa and radish seeds when a combined treatment of hot water (50 °C) + H_2_O_2_ (2%) + acetic acid (0.1%) was applied for a total of 20 min, raising the germination capacity to 94% and 100%, respectively [26]. In this context, Ismaili et al. (2023) [70] indicate that temperature is a critical factor influencing seed dormancy (i.e., the inhibition of germination), as it can either positively or negatively affect this process. Additionally, most species are capable of germinating across a wide temperature range, likely due to the natural conditions they experience, such as day-night temperature fluctuations. Furthermore, the same author notes that high temperatures can break dormancy, whereas low temperatures tend to induce it. Based on the findings of the authors, both in the seeds of the RH variety and the PK variety, the application of a temperature of 50 °C combined with two chemical agents was not only favorable for germination but also likely enhanced this process compared to the use of water at ambient temperature (22 °C) as the control.

Maintaining the germination power of grains is crucial for sprout production as it underlies nutritional, functional, sensory, and technological changes. As outlined by Tuan et al. (2018) [6], the germination process initiates during soaking and concludes with the radicle penetrating the seed layers, encompassing crucial physiological transformations in the grain. While none of the disinfection treatments entirely prevented the germination process in quinoa grains, they did exert discernible effects. Among the disinfectants, alcohol exhibited the most substantial negative impact on radicle characteristics and grain morphology. Ethanol at a concentration of 70% led to radicle shortening and thickness reduction, especially pronounced in the RH variety, suggesting a potentially toxic effect on these [73]. Acetic acid similarly affected radicles, with a more pronounced impact on the RH variety, although to a lesser extent compared to alcohol. According to Kern et al. (2009) [74], ethanol typically stimulates germination in species such as *Oryza sativa*, *Cucumis sativus*, *Avena fatua*, *Lactuca sativa*, *Solanum tuberosum*, and *Avena sativa*. However, in *E. heterophylla*, germination inhibition was observed at concentrations as low as 0.25%, suggesting higher concentrations may cause complete inhibition. This may be due to ethanol prolonging hypoxic conditions and altering cellular respiration, inhibiting ADP phosphorylation in mitochondria, and affecting embryo activity. Similarly, while acetic acid is believed to enhance germination by increasing seed coat permeability, studies on barley, maize, clover, oats, and wheat have shown that it can affect root length, depending on the crop. This occurs during the second germination stage when organic acids affect membrane integrity and energy production, reducing root and shoot elongation, leading to biomass loss, decreased nutrient absorption, and poor sprout development [75].

Organizing the disinfectants based on radicle size and thickness from least to most negative effect yielded the following order for the RH variety: US15 < US30 < 200 ppm SH < HW + H_2_O_2_ + AA < 8% H_2_O_2_ < 4% AA < 70% EtOH. For the PK variety, the order was US15 < US30 < HW + H_2_O_2_ + AA < 200 ppm SH < 8% H_2_O_2_ < 4% AA < 70% EtOH. In both varieties, ultrasound had a positive influence on radicle characteristics, resulting in longer and thicker radicles. Studies on alfalfa, broccoli [76], and peas [68] have demonstrated that ultrasound enhances germination percentages. In peas, ultrasound increased radicle and hypocotyl length by 74% and 34%, respectively. Ultrasound-induced vibrations led to metabolic changes in quinoa grains, stimulating radicle growth. The cavitation generated likely facilitated the rupture of the quinoa seed coat, allowing water and nutrients to pass through. This accelerated protein production and cell division promoted embryo growth and reduced germination time [77,78]. Slight differences between the 15 and 30 min ultrasound treatments could be attributed to potential overdosing, which inversely affects germination [76]. Both ultrasound and hot water stood out as the most effective treatments for stimulating radicle growth in quinoa. Although their mechanisms differ, both induce controlled dormancy disruption by increasing the permeability of the seed coat. Ultrasound generates microbubbles whose implosion causes micro-erosions in the seed tegument, while heat physically softens the coat. These modifications facilitate imbibition, accelerate the onset of germination, and promote more vigorous seedling development [79,80,81].

Disinfection treatments also influenced germination time (data not presented), increasing by up to 20 h compared to the control, which required only 4 h (with no further changes observed thereafter). When sodium hypochlorite was used, the germination time for both varieties was 16 h, while the acetic acid solution required 15 h for the PK variety. As explained earlier, acetic acid could reduce radicle growth, delaying their appearance. However, due to its volatility, it is possible to observe them over time, although not in all seeds [75]. Regarding sodium hypochlorite, it has been shown in rice seeds that although this agent increases both germination capacity and average germination time, beyond certain concentrations (10%), this compound could affect the time required for germination. This could explain the behavior of quinoa seeds [82].

Furthermore, it was observed that the variety influenced both the disinfection treatments and the germination capacity of quinoa grains, suggesting a potential relationship between these factors. The PK variety could have cracks and fissures that provide protection against microbial populations, thereby safeguarding the grain and having a minimal impact on its germination potential. In this context, Tao et al. (2021) [83] highlighted that the complex surface of food products can affect the interaction between disinfectants and the microorganisms present on them. Regarding seed disinfection for sprout production, Beuchat (1997) [45] attributed the limited effectiveness of disinfectant solutions such as calcium hypochlorite, sodium hypochlorite (1800 and 2000 μg/mL of active chlorine), hydrogen peroxide (6%), and ethanol (80%) against *Salmonella* to the presence of crevices and spaces between the cotyledon and the testa in alfalfa seeds.

Various studies have evaluated chemical and physical methods for seed disinfection, showing high antimicrobial efficacy in some cases, although the expected reduction levels are not always achieved. Added to this are limitations related to their practical applicability and, in certain cases, the risk of affecting germination viability. In this context, the disinfection approaches applied in the present study—either through conventional disinfectants or in combination with moderate physical treatments—showed differentiated behaviors depending on the quinoa variety, confirming that although not all treatments achieve optimal reductions, it is possible to obtain significant microbial reduction without compromising germination. This is consistent with previous studies in other species; however, unlike many of those works, the present study adopts a systematic and application-oriented approach that simultaneously evaluates microbial reduction and germination capacity, specifically using Peruvian quinoa varieties. The study highlights the identification of accessible alternatives that allow for balancing microbial reduction with the preservation of germinative capacity in quinoa seeds. This integrated approach aims to produce germinated flours with good microbiological quality, derived from grains that maintained adequate germination performance. This practical strategy, which complements the existing literature, underscores the originality of the work and its relevance in developing disinfection protocols adapted to local contexts and real-world application.

Although the present study focused on microbiological quality and germination, recent works have shown that disinfection treatments can also induce subtle internal changes in seed composition. For example, FTIR and Raman spectroscopy detected conformational modifications in wheat starches after gamma irradiation [84], while high-resolution NMR analysis in lentils treated with plasma-activated water (PAW) revealed the disappearance of sugar signals and an increase in methyl groups in the ^1^H spectrum, confirming internal chemical transformations [85]. Similarly, ultrasound treatments have been associated with increases in phenolic compounds and higher antioxidant activity in hibiscus seeds [86]. These studies suggest that certain treatments may cause biochemical changes that do not affect germination but could have nutritional or functional implications.

To the best of our knowledge, no studies have been published using FTIR or NMR to characterize potential internal changes in quinoa seeds following disinfection procedures, whether chemical or physical. In this regard, future research on this Andean grain should incorporate spectroscopic or molecular analyses to determine whether the applied treatments may induce internal alterations that are not detectable through conventional physiological parameters.

## 4. Conclusions

The application of chemical and combined treatments for disinfecting quinoa grains was assessed in this study. Results revealed that these treatments reduced the population of aerobic mesophilic bacteria and enterobacteria in both quinoa varieties, except in the case of the PK variety, where the reduction was generally lower when alcohol was used. Regarding the use of chemical disinfectants, sodium hypochlorite at a concentration of 200 ppm with an exposure time of 30 min proved to be the most effective treatment in the RH variety (*p* < 0.05), whereas 8% hydrogen peroxide applied for 20 min showed the greatest impact in the PK variety (*p* < 0.05), affecting both mesophilic bacteria and enterobacteria. However, these reductions were not sufficient to consider the resulting flour microbiologically safe. The treatment with 70% ethanol for 10 min was, overall, the least effective in reducing microbial load in both quinoa varieties, and it also notably affected germination capacity.

Among the disinfection methods studied in quinoa grains, the combined use of three barriers (hot water at 50 °C, hydrogen peroxide at 2%, and acetic acid at 0.1%) showed superior efficacy against the evaluated microbial population compared to other treatments. In practical terms, the most effective combined treatment consisted of immersing the previously washed grains in hot water at 50 °C for 10 min, followed by the application of 2% hydrogen peroxide and 0.1% acetic acid for an additional 10 min. To reduce the presence of potential chemical residues, a final rinsing step was included, consisting of two washes with sterile water (≥30 s each), contributing to the overall safety and applicability of the process. The mechanisms by which heat, hydrogen peroxide, and acidic conditions target bacterial cells appear to synergistically enhance disinfection efficacy in both quinoa varieties, resulting in reductions in mesophilic bacterial populations to levels deemed safe for flour production. Moreover, this treatment did not significantly impact the germination power of the grains. Finally, from the perspective of replicating these types of treatments both at an industrial and domestic level, it is important to consider that the concentrations of the chemical agents used, especially sodium hypochlorite and H_2_O_2_ (2%), are quite low, and they could even be slightly increased if necessary to achieve adequate reductions. The increase in costs, whether for the purchase of equipment, energy consumption, or simply the need for an additional chemical agent, is justified by the improvements and primarily the importance of offering a safe product, making these treatments a good alternative. Although physical treatments such as irradiation and ozone have shown high efficacy in seed disinfection, they present limitations relate to infrastructure, safety, and potential effects on viability. In contrast, the liquid methods evaluated in this study constitute a practical, accessible, and safe alternative which is particularly applicable in domestic and semi-industrial contexts.

## Figures and Tables

**Table 1:** Germination power (%) of grains from Rosada de Huancayo (RH) and Pasankalla (PK) quinoa varieties after disinfection treatments.

Treatment Type	Procedure	RH (%)	PK (%)
Control	Untreated quinoa	97.50 ± 3.02 ^a^	99.67 ± 0.52 ^A^
Chemical	200 ppm sodium hypochlorite	100.00 ± 0.00 ^a^	100.00 ± 0.00 ^A^
	8% hydrogen peroxide H_2_O_2_	99.00 ± 0.00 ^a^	100.00 ± 0.00 ^A^
	4% acetic acid	95.67 ± 3.21 ^ab^	100.00 ± 0.00 ^A^
	70% alcohol	91.00 ± 3.00 ^b^	89.67 ± 5.51 ^B^
Combined	Ultrasound 15 min + 200 ppm sodium hypochlorite	100.00 ± 0.00 ^a^	100.00 ± 0.00 ^A^
	Ultrasound 30 min + 200 ppm sodium hypochlorite	99.00 ± 1.00 ^a^	100.00 ± 0.00 ^A^
	Water 50 °C + H_2_O_2_ 2% + Acetic acid 0.1%	98.00 ± 1.73 ^a^	100.00 ± 0.00 ^A^

Values (mean ± SD, n = 3) within each column followed by different superscript letters are significantly different (*p* < 0.05). Lowercase letters (a, b, ab) indicate differences within RH (%), while uppercase letters (A, B) indicate differences within PK (%).

## Data Availability

The original contributions presented in this study are included in the article; further inquiries can be directed to the corresponding author.

## Abbreviations

The following abbreviations are used in this manuscript:

RH	Rosada de Huancayo quinoa variety
PK	Pasankalla quinoa variety
200 ppm SH	200 ppm sodium hypochlorite
4% AA	4% acetic acid
70% EtOH	70% ethanol
8% H_2_O_2_	8% hydrogen peroxide
HW + H_2_O_2_ + AA	Hot water (50 °C, 10 min) followed by immersion in hydrogen peroxide (2% *v*/*v*) and acetic acid (0.1% *v*/*v*)
US15	Ultrasound treatment for 15 min in water with 200 ppm sodium hypochlorite
US30	Ultrasound treatment for 30 min in water with 200 ppm sodium hypochlorite
